# Biologic Disease-Modifying Antirheumatic Drugs for Preventing Radiographic Progression in Psoriatic Arthritis: A Systematic Review and Network Meta-Analysis

**DOI:** 10.3390/pharmaceutics14102140

**Published:** 2022-10-08

**Authors:** Szu-Hsuan Wang, Chia-Ling Yu, Tzu-Yu Wang, Chung-Han Yang, Ching-Chi Chi

**Affiliations:** 1 Department of Pharmacy, Chang Gung Memorial Hospital, Linkou, Taoyuan 33305, Taiwan; 2 Department of Applied Cosmetology, Lee-Ming Institute of Technology, New Taipei 24346, Taiwan; 3 Division of Rheumatology, Allergy and Immunology, Chang Gung Memorial Hospital, Linkou, Taoyuan 33305, Taiwan; 4 Department of Dermatology, Chang Gung Memorial Hospital, Linkou, Taoyuan 33305, Taiwan; 5 School of Medicine, College of Medicine, Chang Gung University, Taoyuan 33302, Taiwan

**Keywords:** psoriatic arthritis, radiographic progression, biologic, biologic disease-modifying antirheumatic drugs (bDMARDs)

## Abstract

The prevention of joint deformity is among the most important treatment goals of psoriatic arthritis. Some biologics disease-modifying antirheumatic drugs (bDMARDs) have been demonstrated to be effective for both the skin and joints, as well as for slowing radiographic progression. However, there has been a lack of direct comparisons of bDMARDs. To evaluate the comparative effects of bDMARDs in preventing radiographic progression in psoriatic arthritis, we conducted a systematic review and network meta-analysis. On March 7 2022, a search for relevant randomized trials was conducted on MEDLINE, Embase, and the Cochrane Central Register of Controlled Trials. Our outcomes included radiographic non-progression, a mean change in the total radiographic score, and adverse events leading to discontinuation (DAE) at week 24. We included 11 trials on 10 bDMARDs, involving 4010 participants. Most bDMARDs were more effective than placebos in achieving radiographic non-progression, including adalimumab (odds ratio (OR) 4.7, 95% confidence interval (CI) 2.66–8.29), etanercept (OR 4.19, 95% CI 1.65–10.61), certolizumab pegol (OR 2.83, 95% CI 1.55–5.2), secukinumab 300 mg (OR 2.63, CI 1.62–4.27), infliximab (OR 2.54, CI 1.13–5.69), ixekizumab (OR 2.22, 95% CI 1.06–4.65), golimumab (OR 2.21, 95% CI 1.24–3.93), and abatacept (OR 1.54, 95% CI 1.03–2.28). A significant reduction in the total radiographic score was found in infliximab (standardized mean difference (SMD) −0.59, 95% CI −0.87, −0.3), etanercept (SMD −0.51, 95% CI −0.78, −0.23), adalimumab (SMD −0.45, 95% CI −0.64, −0.26), ixekizumab (SMD −0.37, 95% CI −0.62, −0.12), secukinumab 300 mg (SMD −0.33, 95% CI −0.50, −0.15), golimumab (SMD −0.33, 95% CI −0.58, −0.09), secukinumab 150 mg (SMD −0.25, 95% CI −0.43, −0.07), certolizumab pegol (SMD −0.23, 95% CI −0.44, −0.03), and ustekinumab (SMD −0.19, 95% CI −0.35, −0.33). No significant differences in DAE were detected between bDMARDs. In conclusion, anti-tumor necrosis factor agents (adalimumab, infliximab, and etanercept) may be preferred for treating psoriatic arthritis for their superiority in preventing radiographic progression.

## 1. Introduction

Psoriatic arthritis is a chronic inflammatory arthritis found in up to approximately 20% of patients with psoriasis, 25% of those with moderate to severe psoriasis [1], and 0.1 to 0.25% of the general population [2]. Psoriatic arthritis is characterized by peripheral arthritis, enthesitis, dactylitis, spondylitis, and psoriasis of the skin and nails [3,4,5]. Moreover, persistent inflammation may damage cartilage and bone, leading to bone erosions and joint space narrowing, soft tissue changes, and total joint destruction, which are detected and characterized radiographically [6,7,8]. Progressive joint damage has been reported in over half of patients with psoriatic arthritis, which is often associated with functional impairment and disability [9]. Although the time course of radiographic progression varies widely, almost half of patients exhibit structural damage and functional impairment within 2 years of developing symptoms [10]. The risk of death is increased in patients with psoriatic arthritis compared to the general population, and the severity of psoriatic arthritis at presentation is a predictor of mortality [11]. The assessment of radiographic disease progression in psoriatic arthritis is a measure of disease severity and the effect of treatment on disease progression [12]. Treatment recommendations state that, in addition to low disease activity, goals of treatment are to prevent structural damage and optimize patient functioning and quality of life [8,13].

Evaluating the progression of structural damage has become important in clinical trials that evaluate treatments for psoriatic arthritis. Treatments recommended for psoriatic arthritis include conventional synthetic disease-modifying antirheumatic drugs (csDMARDs), biologic agents (bDMARDs), Janus kinase inhibitors, and phosphodiesterase-4 inhibitors [8,13,14]. csDMARDs have not been demonstrated to be effective in inhibiting structural damage; in contrast, there are plentiful trials of bDMARDs which demonstrate good efficacy for both the skin and joints, as well as slowing or halting radiographic progression [15,16,17,18,19,20,21,22,23,24,25,26,27]. These bDMARDs include five available anti-tumor necrosis factor (TNF) agents (etanercept, infliximab, adalimumab, golimumab, and certolizumab pegol), one interleukin (IL)-12/23 inhibitor (ustekinumab), one p19 subunit of IL-23 inhibitor (guselkumab), two IL-17A inhibitors (secukinumab, ixekizumab), and one selective T-cell costimulation modulator (abatacept). 

A previous meta-analysis which only included anti-TNF agents found better effects in preventing radiographic progression when compared to the placebo [28]. The efficacy of anti-TNF agents, IL inhibitors, and abatacept may retard radiographic progression in psoriatic arthritis patients compared with placebo; however, this meta-analysis did not include guselkumab and lacked comparisons between different bDMARDs [29]. A recent NMA used guselkumab instead of a placebo as the comparator and did not examine the comparative effects between different bDMARDs [30]. As there has been a lack of direct comparisons between the effect of different bDMARDs on radiographic structural damage in psoriatic arthritis, and given that joint deformity may cause disability in affected patients, we aimed to examine the comparative effects of bDMARDs in preventing radiographic progression in psoriatic arthritis.

## 2. Materials and Methods

We conducted a systematic review and network meta-analysis (NMA) to evaluate the comparative efficacy and safety of US Food and Drug Administration (FDA)-approved bDMARDs in preventing radiographic structural damage in psoriatic arthritis. The study was conducted in accordance with the Preferred Reporting Items for Systematic Reviews and Meta-Analyses for Network Meta-Analyses (PRISMA-NMA) [31]. This study protocol was registered on International Prospective Register of Systematic Reviews (PROSPERO) (CRD42021233381). This study was exempted from ethics review by the Chang Gung Medical Foundation (202002102B1).

### 2.1. Literature Search

We identified relevant randomized controlled trials (RCTs) by searching the MEDLINE, the Cochrane Central Register of Controlled Trials (CENTRAL), and Embase databases from inception to 7 March 2022. The search terms included “randomized controlled trial”, “radiographic progression”, “Sharp score”, “psoriatic arthritis”, and medicines of interest (including etanercept, adalimumab, infliximab, golimumab, certolizumab pegol, ustekinumab, secukinumab, ixekizumab, brodalumab, guselkumab, risankizumab, clazakizumab, and ABT-122). We also scanned the bibliographies of relevant reviews. The detailed search strategy is presented in Table S1, Supplementary Material.

### 2.2. Study Selection

Two researchers (SSW and CLY) independently selected relevant studies that met the following inclusion criteria: (1) used a randomized controlled trial (RCT); (2) assessed the effects of bDMARDs in preventing joint deformity in psoriatic arthritis; and (3) reported data on the radiographic progression of joints at week 24. Studies were excluded (1) if there was not an RCT and (2) if there was a lack of usable data. We evaluated the titles and abstracts of the retrieved literature. If the abstract did not provide enough information for inclusion or exclusion, eligibility was confirmed by a full-text evaluation. Any discrepancies in study selection were resolved by discussion with a third researcher (CCC).

### 2.3. Data Extraction and Risk of Bias Assessment

The extracted information from each study included publication year, authors, interventions and regimens, the number of participants, age, baseline radiographic score, psoriatic arthritis duration, tender joint count (TJC), swollen joint count (SJC), and C-reactive protein (CRP). Our outcomes of interest included radiographic evidence of non-progression, the mean change in the total radiographic score, and discontinuations due to adverse events at week 24. We extracted radiographic data on the modified Sharp score or the modified Sharp–van der Heijde method for psoriatic arthritis. Both scores measure bone erosion and joint space narrowing for hands, wrists, and feet [32]. Non-progression was defined as a change from the baseline in the total radiographic score ≤ 0 or ≤ 0.5. The Cochrane Collaboration tool was used to evaluate the risk of bias for included RCTs [33].

### 2.4. Statistical Analysis

We combined direct and indirect evidence by using a frequentist NMA [34]. Only data on FDA-approved regimens and placebo were included for NMA. We implemented a fixed-effects model to calculate pooled analyses since, in most cases, the treatment of interest was evaluated in one trial, and the number of included trials per comparison was too small to estimate between-study heterogeneity [35]. We used odds ratio (OR) with a 95% confidence interval (CI) to express binary outcome data. As clinical trials on etanercept and adalimumab used a modified Sharp score to assess the effects on structural damage [16,17], while other trials used a modified Sharp–van der Heijde method, we used standardized mean difference (SMD) with a 95% CI to pool the mean change in the total radiographic score from different scales [36].

We calculated the probability of efficacy rankings measured by the surface under the cumulative ranking curve (SUCRA) for each intervention [37]. The higher the SUCRA value, the higher the likelihood that the intervention was in the top rank [38]. Publication bias was assessed by funnel plots and Egger’s test [36]. We used two-dimensional plots to obtain a meaningful grouping of treatment [39,40]. The statistical analyses for the NMA were performed by using the Stata version 15.1 (College Station, TX, USA).

## 3. Results

### 3.1. Search Results and Study Characteristics

**Figure 1:**
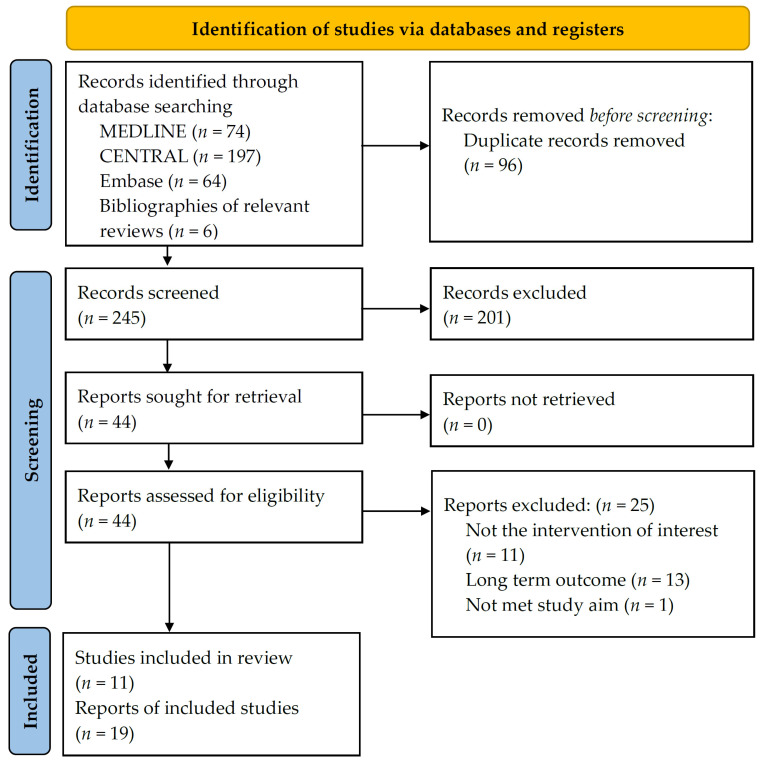
PRISMA study flow chart.

Our search identified a total 341 records. After removing duplicates, 244 records were screened by title and abstract, leaving 41 articles for full-text assessment (Figure 1). Six additional articles were identified by screening the references of relevant reviews. A total of 11 RCTs on 10 bDMARDs, involving 4010 participants [16,18,20,21,23,24,25,26,41,42], were included for this systemic review. 

The characteristics of the included studies are summarized in Table 1. The mean age of participants ranged from 44.9 to 51 years. The average duration of psoriatic arthritis ranged from 3.4 to 9.8 years. The mean tender joint count ranged from 18 to 25.8 and the mean swollen joint count ranged from 9.9 to 14.4. The risk of bias assessment is summarized in Figure S1. None of the included RCTs were rated with a high risk of bias.

### 3.2. Overall Geometric Structure of the Whole Network

**Figure 2:**
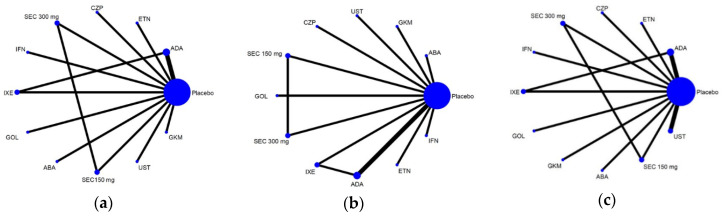
Geometric structure for efficacy and safety. (**a**) Radiographic non-progression; (**b**) change in the total radiographic score; (**c**) discontinuation due to adverse events. PBO: placebo; IFN: infliximab; ADA: adalimumab; UST: ustekinumab; GOL: golimumab; ABA: abatacept; SEC: secukinumab; CZP: certolizumab pegol; ETN: etanercept; IXE: ixekizumab; GKM: guselkumab.

**Figure 3:**
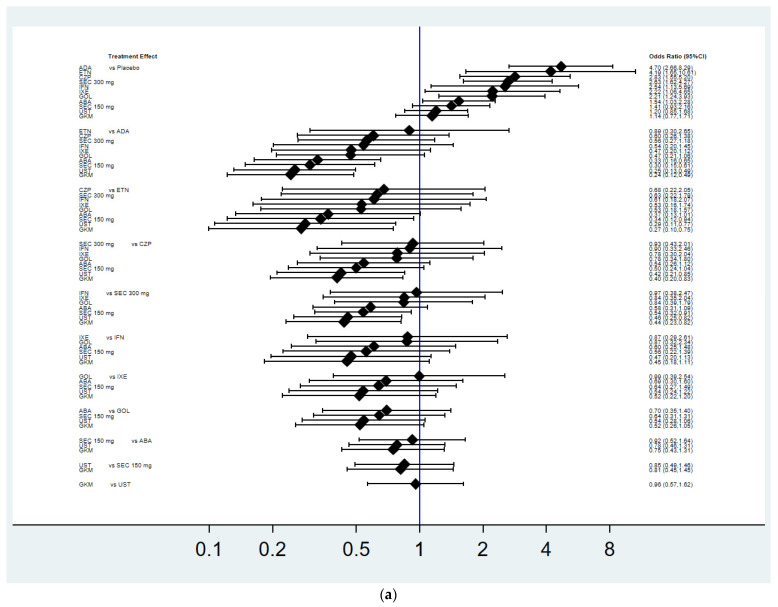

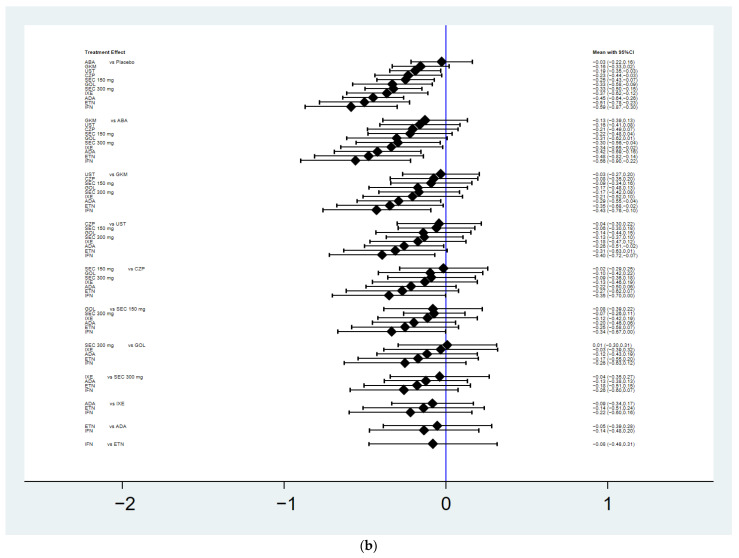

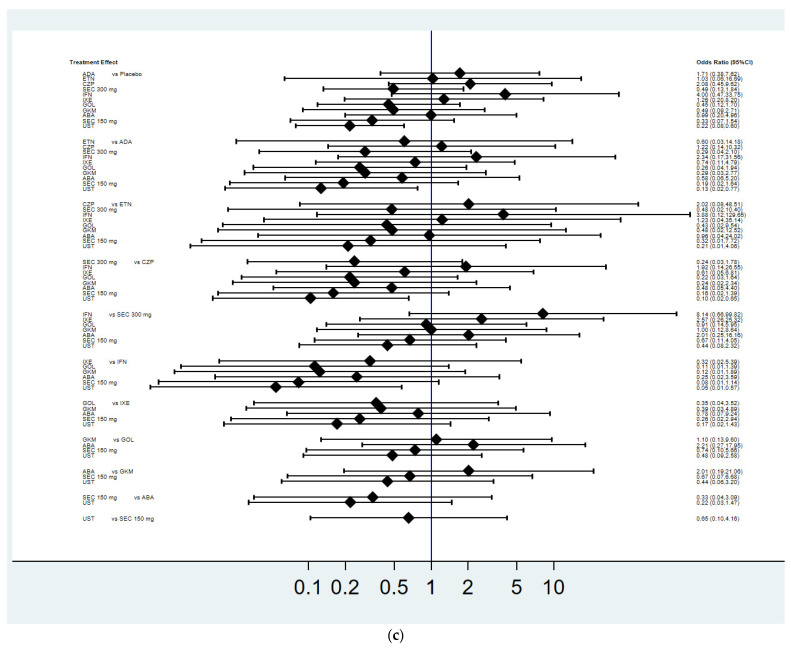
Network meta-analysis for efficacy and safety. (**a**) Radiographic non-progression; (**b**) change in total radiographic score; (**c**) discontinuation due to adverse events. PBO: placebo; IFN: infliximab; ADA: adalimumab; UST: ustekinumab; GOL: golimumab; ABA: abatacept; SEC: secukinumab; CZP: certolizumab pegol; ETN: etanercept; IXE: ixekizumab; GKM: guselkumab.

There were 13 pairwise comparisons that included 11 treatments, 10 drugs with placebo in the NMA for the non-progression of structure damage (Figure 2a), the mean change in the total radiographic score (Figure 2b), and adverse events leading to discontinuation (DAE) (Figure 2c) (infliximab, adalimumab, ustekinumab, golimumab, abatacept, secukinumab 300 mg, secukinumab 150 mg, certolizumab pegol, etanercept, ixekizumab, guselkumab, and placebo). The effect sizes of the NMA are summarized in Figure 3, and the surface under cumulative ranking curve (SUCRA) rankings are detailed in Table 2.

### 3.3. Achievement of Radiographic Non-Progression

As shown in Figure 3a and Table S2, the NMA found that, when compared with placebo, adalimumab (OR 4.7, 95% CI 2.66–8.29), etanercept (OR 4.19, 95% CI 1.65–10.61), certolizumab pegol (OR 2.83, 95% CI 1.55–5.2), secukinumab 300 mg (OR 2.63, CI 1.62–4.27), infliximab (OR 2.54, CI 1.13–5.69), ixekizumab (OR 2.22, 95% CI 1.06–4.65), golimumab (OR 2.21, 95% CI 1.24–3.93), and abatacept (OR 1.54, 95% CI 1.03–2.28) were more effective in achieving radiographic non-progression at week 24. However, there were no significant differences in achieving radiographic non-progression for secukinumab 150 mg, ustekinumab, and guselkumab when compared with placebo. Regarding the ranking of treatment efficacy, adalimumab (SUCRA 92.8%) was associated with the greatest treatment for achieving radiographic non-progression, followed by etanercept (SUCRA 85.9%).

### 3.4. Mean Change in the Total Radiographic Score

The NMA found that, except for abatacept, infliximab (SMD −0.59, 95% CI −0.87, −0.3), etanercept (SMD −0.51, 95% CI −0.78, −0.23), adalimumab (SMD −0.45, 95% CI −0.64, −0.26), ixekizumab (SMD −0.37, 95% CI −0.62, −0.12), secukinumab 300 mg (SMD −0.33, 95% CI −0.50, −0.15), golimumab (SMD −0.33, 95% CI −0.58, −0.09), secukinumab 150 mg (SMD −0.25, 95% CI −0.43, −0.07), certolizumab pegol (SMD −0.23, 95% CI −0.44, −0.03), and ustekinumab (SMD −0.19, 95% CI−0.35, −0.33) were more effective than the placebo in reducing the total radiographic score for structural damage (Figure 3b and Table S3). Additionally, the analysis on SUCRA showed infliximab (SUCRA 91.7%) ranked the best in reducing the total radiographic score, followed by etanercept (SUCRA 82.6%).

### 3.5. Safety

Most bDMARDs did not significantly differ from placebo in DAE, but ustekinumab was associated with lowered odds for DAE when compared with adalimumab, certolizumab pegol, infliximab, and placebo (Figure 3c and Table S4). Based on SUCRA values, a larger SUCRA value indicated safer treatment. Ustekinumab (SUCRA 88.6%) was the best in terms of safety, followed by secukinumab 150 mg (SUCRA 75.3%) and golimumab (SUCRA 68.9%).

### 3.6. Ranking Plot of Different Treatments

**Figure 4:**
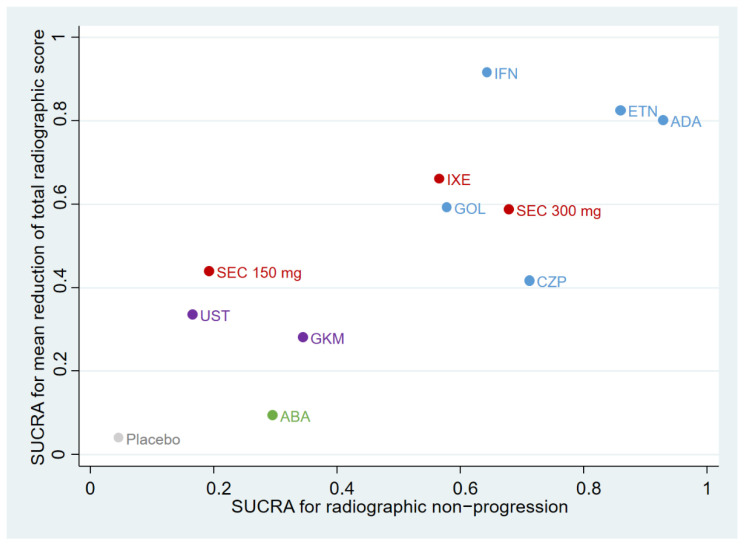
Ranking plot of different biological treatment for psoriasis arthritis patient network. Based on analysis of surface under the cumulative ranking curve (SUCRA) values for two different outcomes: non-progression and mean reduction in the total of radiographic score. Treatments in the upper right corner were associated with achieving both outcomes.

As shown in Figure 4, the ranking plot was based on SUCRA values for two different outcomes: radiographic non-progression and a mean reduction in the total radiographic score. The interventions were divided into four clusters according to their pharmacologic effects. The group in blue comprised anti-TNF agents (infliximab, etanercept, adalimumab, golimumab, and certolizumab pegol); the group in red comprised IL-17A inhibitors (ixekizumab and secukinumab); the group in purple comprised the IL-12/23 inhibitor (ustekinumab) and the p19 subunit of the IL-23 inhibitor (guselkumab); and the group in green comprised the selective T-cell costimulation modulator inhibitor (abatacept). Adalimumab, infliximab, and etanercept were the top three treatments after considering both efficacy outcomes.

### 3.7. Publication Bias

Accordingly, funnel plots of publication bias across the included studies (Figures S2–S4) revealed general symmetry, and the results of Egger’s test indicated no significant publication bias among the articles included in the NMA (Figures S5–S7). Publication bias and small study effects were not found in the outcomes of radiographic non-progression, the mean change in the total radiographic score, and DAE.

## 4. Discussion

This study is a network meta-analysis examining the effects of FDA-approved bDMARD regimens in preventing joint deformity in psoriatic arthritis. Network meta-analysis is a technique used for comparing multiple treatments using both direct and indirect comparisons across trials, and is beneficial for ranking treatments [39]. Due to the lack of direct comparisons between various bDMARDs, we employed a network meta-analysis to calculate the comparative effects on radiographic structural damage in psoriatic arthritis. We found that most bDMARDs were more effective than the placebo in achieving radiographic non-progression at week 24, except for secukinumab 150 mg, ustekinumab, and guselkumab. Most bDMARDs reduced the total radiographic score at week 24, except for abatacept and guselkumab. Meanwhile, most bDMARDs did not differ from placebo in discontinuation, due to adverse events. We further ranked bDMARDs based on the SUCRA for achieving radiographic non-progression and a reduction in the total radiographic score. The top three treatments in preventing join deformity were adalimumab, infliximab, and etanercept. The present NMA filled in the knowledge gap and examined the comparative efficacy and safety of various bDMARDs, including anti-TNF agents, IL inhibitors, and abatacept.

In our meta-analysis, anti-TNF agents showed better performance in preventing radiographic progression than IL inhibitors. Araujo et al. found that synovial component is less sensitive to IL-12/23 inhibition, while the IL-12/23 inhibitor primarily targets the enthesitis and skin disease [43]. Boutet et al. suggested that anti-TNF agents have similar rates of response in the skin and joints, while IL-17 inhibitors have lower efficacy for joint response, but a higher response rate for skin lesions [44]. The exact mechanism for a better radiographic outcome in patients treating with anti-TNF agents is still unclear. The pathogenesis of psoriatic arthritis is related to the innate and adaptive immune response involving different proinflammatory cytokine, including TNF, IL-1β, IL-6, IL-22, IL-23, IL-17A, and IL-18 [45]. The psoriatic gene expression patterns in the skin and joints are different, revealing lower IL-17 gene expression in the joints than in the skin and comparable TNF and interferon gamma gene expression in both tissues [46]. Further studies are needed to confirm that cytokine genetic divergence of the joint and skin lesions in psoriatic arthritis can account for different response to various biologics.

Despite there being different definitions of active psoriatic arthritis between studies, the baseline characteristics (e.g., age, psoriatic arthritis duration, and baseline radiographic score) of their populations were similar. The loss to follow-up rate was very low in our included trials (0–3.4%) [47]. For four bDMARDs (secukinumab, ustekinumab, abatacept, and certolizumab pegol) in studies [20,23,24,42], participants (17% to 62%) were allowed to have previous exposure to anti-TNF agents (Table 1). However, one previous study found that prior anti-TNF agents might not influence the radiographic efficacy of IL inhibitors [29]. 

Our study has some limitations. First, most of our included trials were conducted in North America and Europe, while only three included trials involved Asian participants [25,26,42]. There is still a lack of adequate data to inform how different ethnic groups may respond to the same treatment. Second, there was only one trial reporting radiographic outcome data for each bDMARD regimen, with a limited sample size of <400 [48]. Third, all included trials only assessed erosions and joint space narrowing of peripheral joints, but did not assess anabolic bone formation (i.e., structural changes associated with enthesitis [49]) or progression measures in the axial skeleton [50]). Fourth, we were unable to analyze the effects of some newly available bDMARDs because of lacking relevant data. For example, radiographic progression was not assessed in the pivotal trails on ABT122, brodalumab, clazakizumab, and risankizumab. 

## 5. Conclusions

In conclusion, bDMARDs may inhibit radiographic progression in psoriatic arthritis patients compared with placebos. This study found three anti-TNF agents (adalimumab, infliximab, and etanercept) may be the preferred treatments for psoriatic arthritis for their superiority in achieving radiographic non-progression and reducing the total radiographic score.

## Figures and Tables

**Table 1:** Baseline characteristics of included trials.

Study	Treatment	*n*	Prior bDMARD use (%)	Age (years)	Baseline Radiographic Score	Duration of Psoriatic Arthritis (Years)	Tender Joint Count	Swollen Joint Count	C-Reactive Protein (mg/dL)
**Anti-tumor necrosis factor agents**									
Gladman (2007)	Adalimumab 40 mg Q2W	151	0%	48.6 ± 12.5	22.7 ± 46	9.8 ± 8.3	23.9 ± 17.3	14.3 ± 12.2	1.4 ± 2.1
ADEPT	Placebo	162	0%	49.2 ± 11.1	19.1 ± 35.5	9.2 ± 8.7	25.8 ± 18	14.3 ± 11.1	1.4 ± 1.7
van der Heijde (2014)	Certolizumab pegol 200 mg Q2W	138	22.50%	48.2 ± 12.3	18.0 ± 30.6	9.6 ± 8.5	21.5 ± 15.3	11.0 ± 8.8	0.87 (0.01–8.70)
RAPID-PSA	Certolizumab pegol 400 mg Q4W	135	17%	47.1 ± 10.8	22.8 ± 46.5	8.1 ± 8.3	19.6 ± 14.8	10.5 ± 7.5	0.70 (0.02–23.80)
	Placebo	136	19.10%	47.3 ± 11.1	24.4 ± 49.7	7.9 ± 7.7	19.9 ± 14.7	10.4 ± 7.6	0.90 (0.02–13.10)
Mease (2006)	Etanercept 25 mg BIW	101	0%	47.6	25.89	9.0	Not reported	Not reported	Not reported
	Placebo	104	0%	47.3	18.30	9.2	Not reported	Not reported	Not reported
Kavanaugh (2012)	Golimumab 50 mg Q4W	146	0%	45.7 ± 10.7	23.85 ± 35.41	7.2 ± 6.8	24.0 ± 17.1	14.1 ± 11.4	1.3 ± 1.6
GO-REVEAL	Placebo	113	0%	47.0 ± 10.6	18.15 ± 27.76	7.6 ± 7.9	21.9 ± 14.7	13.4 ± 9.8	1.3 ± 1.6
van der Heijde (2007)	Infliximab 5 mg/kg Q8W	100	0%	47.1 ± 12.8	30.3 ± 61.4	8.4 ± 7.2	24.6 ± 14.1	13.9 ± 7.9	1.9 ± 2.1
IMPACT 2	Placebo	100	0%	46.5 ± 11.3	39.1 ± 82.8	7.5 ± 7.8	25.1 ± 13.3	14.4 ± 8.9	2.3 ± 3.4
**IL-17A inhibitors**									
Mease (2017)	Ixekizumab 80 mg Q4W	107	0%	49.1 ± 10.1	19.2 ± 32.7	6.2 ± 6.4	20.5 ± 13.7	11.4 ± 8.2	1.28 ± 1.64
SPIRIT-P1	Adalimumab 40 mg Q2W	101	0%	48.6 ± 12.4	15.9 ± 27.4	6.9 ± 7.5	19.3 ± 13.0	9.9 ± 6.5	1.32 ± 1.91
	Placebo	106	0%	50.6 ± 12.3	17.6 ± 28.6	6.3 ± 6.9	19.2 ± 13.0	10.6 ± 7.3	1.51 ± 2.36
Mease (2018)	Secukinumab 300 mg (LD) Q4W	222	30.70%	48.9 ± 12.8	12.9 ± 23.7	6.7 ± 8.3	19.8 ± 15.1	10.0 ± 8.0	Not reported
FUTURE 5	Secukinumab 150 mg (LD) Q4W	220	29.50%	48.4 ± 12.9	13.6 ± 25.9	6.7 ± 7.1	21.2 ± 15.9	12.1 ± 10.5	Not reported
	Placebo	332	29.50%	49.0 ± 12.1	15 ± 38.2	6.6 ± 7.6	21.2 ± 16.2	11.7 ± 10.8	Not reported
**p19 subunit of IL-23 inhibitor**									
Mease (2020)	Guselkumab 100 mg Q8W	248	0%	44.9 ± 11.9	23.0 ± 37.8	5.1 ± 5.5	19.8 ± 11.9	11.7 ± 6.8	1.3 (0.7–2.5)
DISCOVER-2	Placebo	246	0%	46.3 ± 11.7	23.8 ± 37.8	5.8 ± 5.6	21.6 ± 13.1	12.3 ± 6.9	1.2 (0.5–2.6)
**IL-12/23 inhibitor**									
Kavanaugh (2014)	PSUMMIT-1: Ustekinumab 45 mg Q12W	205	0%	48.0 (39.0–55.0)	30.1 ± 51.7	3.4 (1.2–9.2)	18.0 (12.0–28.0)	10.0 (7.0–15.0)	1.00 (0.59–2.11)
	PSUMMIT-1: Placebo	206	0%	48.0 (39.0–57.0)	29.9 ± 59.3	3.6 (1.0–9.7)	22.0 (13.0–33.0)	12.0 (8.0–19.0)	0.96 (0.60–1.86)
	PSUMMIT-2: Ustekinumab 45 mg Q12W	103	58.25%	49.0 (40.0–56.0)	31.1 ± 48.9	5.3 (2.3–12.2)	22.0 (15.0–33.0)	12.0 (8.0–19.0)	1.30 (0.45–3.63)
	PSUMMIT-2: Placebo	104	59.61%	48.0 (38.5–56.0)	24.3 ± 48.0	5.5 (2.3–12.2)	21.0 (11.0–30.0)	11.0 (7.0–18.0)	0.85 (0.46–2.20)
**Selective T-cell costimulation modulator**									
Mease (2017)	Abatacept 125 mg QW	213	60.60%	51.0 ± 10.7	20.0 ± 46.8	8.3 ± 8.1	21.0 ± 13.4	12.1 ± 7.8	1.40 ± 2.09
ASTRAEA	Placebo	211	61.60%	49.8 ± 11.3	17.7 ± 39.6	8.8 ± 8.3	19.3 ± 13.1	11.1 ± 7.2	1.43 ± 3.03

bDMARD: biologics disease-modifying antirheumatic drugs; IL: interleukin; LD: loading dose; QW: weekly; Q2W: every 2 weeks; Q4W: every 4 weeks; Q8W: every 8 weeks; Q12W: every 12 weeks. Continuous variables are presented as mean ± SD when appropriate.

**Table 2:** Ranking of treatments according to SUCRAs.

Intervention	Efficacy		Safety DAE (%)
	Non-Progression (%)	Total Sharp Score (%)	
**ADA**	92.8	80.2	27.8
**ETN**	85.9	82.6	44.9
**CZP**	71.1	41.7	23.7
**SEC 300 mg**	67.8	58.8	66.4
**IFN**	64.2	91.7	13.9
**IXE**	56.5	66.2	37.7
**GOL**	57.7	59.3	68.9
**GKM**	34.4	28.1	67.2
**ABA**	29.5	9.5	43.9
**SEC 150 mg**	19.2	44	75.3
**UST**	16.5	33.6	88.6
**PBO**	4.5	4.1	41.7

ABA: abatacept; ADA: adalimumab; CZP: certolizumab pegol; DAE: discontinuation due to an adverse event; ETN: etanercept; GKM: guselkumab; GOL: golimumab; IFN: infliximab; IXE: ixekizumab; PBO: placebo; SEC: secukinumab; SUCRA: surface under the cumulative ranking curve; UST: ustekinumab.

## Data Availability

All data are incorporated into the article and its online supplementary material.

## Supplementary Materials

The following supporting information can be downloaded at: https://www.mdpi.com/article/10.3390/pharmaceutics14102140/s1. Table S1: Search strategy. Table S2: League table of treatment response: analysis based on radiographic non-progression. Table S3: League table of treatment response: analysis based on changes in the total radiographic score. Table S4: League table of treatment response: analysis based on discontinuation due to adverse events of psoriatic arthritis patient receiving bDMARDs. Figure S1: Risk of bias, presented with the Cochrane Collaboration’s Assessment Tool. Figure S2: Funnel plot for radiographic non-progression of psoriatic arthritis patient receiving bDMARDs. Figure S3: Funnel plot for changes in the total radiographic score of psoriatic arthritis patient receiving bDMARDs. Figure S4: Funnel plot for discontinuation due to adverse events of psoriatic arthritis patient receiving bDMARDs. Figure S5: Egger’s test for radiographic non-progression. Figure S6: Egger’s test for change in the total radiographic score. Figure S7: Egger’s test for discontinuation due to adverse events.
