# Supplementary material for: Biologic Disease-Modifying Antirheumatic Drugs for Preventing Radiographic Progression in Psoriatic Arthritis: A Systematic Review and Network Meta-Analysis
Source: Pharmaceutics. 2022 Oct 8;14(10):2140. doi: 10.3390/pharmaceutics14102140 (PMC9608970; doi:10.3390/pharmaceutics14102140)

Table S1. Search strategy

**Medline search strategy**
#1. randomized controlled trial.pt.
#2. controlled clinical trial.pt.
#3. randomized.ab.
#4. placebo.ab.
#5. clinical trials as topic.sh.
#6. randomly.ab.
#7. trial.ti.
#8. 1 or 2 or 3 or 4 or 5 or 6 or 7
#9. exp animals/ not humans.sh.
#10. 8 not 9
#11. exp Arthritis, Psoriatic/ or psoriatic arthritis.mp.
#12. etanercept.mp.
#13. adalimumab.mp.
#14. infliximab.mp.
#15. golimumab.mp.
#16. certolizumab pegol.mp.
#17. ustekinumab.mp.
#18. secukinumab.mp.
#19. ixekizumab.mp.
#20. brodalumab.mp.
#21. guselkumab.mp.
#22. risankizumab.mp.
#23. clazakizumab.mp.
#24. abatacept.mp.
#25. ABT-122.mp.
#26. or/12-25
#27. Sharp score.mp.
#28. radiographic progression.mp.
#29. 27 or 28
#30. 10 and 11 and 26 and 29
**CENTRAL search strategy**
#1 (psoriatic arthritis) (Word variations have been searched)
#2 MeSH descriptor: [Arthritis, Psoriatic] explode all trees
#3 #1 OR #2
#4 (etanercept)

#5 (adalimumab)
#6 (infliximab)
#7 (golimumab)
#8 (certolizumab pegol)
#9 (ustekinumab)
#10 (secukinumab)
#11 (ixekizumab)
#12 (brodalumab)
#13 (guselkumab)
#14 (risankizumab)
#15 (clazakizumab)
#16 (abatacept)
#17 (ABT-122)
#18 #4 OR #5 OR #6 OR #7 OR #8 OR #9 OR #10 OR #11 OR #12 OR #13 OR #14 OR #15 OR #16 OR #17
#19 Sharp score
#20 radiographic progression
#21 #19 OR #20
#22 #3 AND #18 AND #21

EMBASE search strategy

#1. randomized AND controlled AND trial:pt
#2. controlled AND clinical AND trial:pt
#3. randomized:ab
#4. placebo:ab
#5. 'clinical trial (topic)'
#6. randomly:ab
#7. trial:ti
#8. #1 OR #2 OR #3 OR #4 OR #5 OR #6 OR #7
#9. 'animals'/exp NOT 'humans'/de
#10. 8 NOT 9
#11. 'psoriatic arthritis'/exp OR (psoriatic AND arthritis)
#12. Etanercept
#13. Adalimumab
#14. Infliximab
#15. Golimumab
#16. certolizumab AND pegol
#17. Ustekinumab
#18. Secukinumab

#19. Ixekizumab
#20. Brodalumab
#21. Guselkumab
#22. Risankizumab
#23. Clazakizumab
#24. Abatacept
#25. 'abt 122'
#26. #12 OR #13 OR #14 OR #15 OR #16 OR #17 OR #18 OR #19 OR #20 OR #21 OR #22 OR #23 OR #24 OR #25
#27. sharp AND score
#28. radiographic AND progression
#29. #27 OR #28
#34. #10 AND #11 AND #26 AND #29

Table S2. League table of treatment response: analysis based on radiographic non-progression. The effect sizes are reported as odds ratio and 95% confidence intervals. Comparisons between treatments should be read from right to left.

Placebo	4.70 (2.66,8.29)	4.19 (1.65,10.61)	2.83 (1.55,5.20)	2.63 (1.62,4.27)	2.54 (1.13,5.69)	2.22 (1.06,4.65)	2.21 (1.24,3.93)	1.54 (1.03,2.28)	1.41 (0.93,2.16)	1.20 (0.85,1.68)	1.14 (0.77,1.71)
0.21 (0.12,0.38)	ADA	0.89 (0.30,2.65)	0.60 (0.26,1.38)	0.56 (0.27,1.18)	0.54 (0.20,1.45)	0.47 (0.20,1.12)	0.47 (0.21,1.06)	0.33 (0.16,0.65)	0.30 (0.15,0.61)	0.25 (0.13,0.49)	0.24 (0.12,0.49)
0.24 (0.09,0.60)	1.12 (0.38,3.33)	ETN	0.68 (0.22,2.05)	0.63 (0.22,1.79)	0.61 (0.18,2.07)	0.53 (0.16,1.74)	0.53 (0.18,1.57)	0.37 (0.13,1.01)	0.34 (0.12,0.94)	0.29 (0.11,0.77)	0.27 (0.10,0.75)
0.35 (0.19,0.65)	1.66 (0.72,3.80)	1.48 (0.49,4.48)	CZP	0.93 (0.43,2.01)	0.90 (0.33,2.46)	0.78 (0.30,2.04)	0.78 (0.34,1.80)	0.54 (0.26,1.12)	0.50 (0.24,1.04)	0.42 (0.21,0.85)	0.40 (0.20,0.83)
0.38 (0.23,0.62)	1.79 (0.85,3.77)	1.59 (0.56,4.55)	1.08 (0.50,2.34)	SEC 300 mg	0.97 (0.38,2.47)	0.84 (0.35,2.04)	0.84 (0.39,1.79)	0.58 (0.31,1.09)	0.54 (0.32,0.91)	0.46 (0.25,0.82)	0.44 (0.23,0.82)
0.39 (0.18,0.88)	1.85 (0.69,4.97)	1.65 (0.48,5.65)	1.12 (0.41,3.06)	1.04 (0.40,2.65)	IFN	0.87 (0.29,2.61)	0.87 (0.32,2.34)	0.60 (0.25,1.48)	0.56 (0.22,1.39)	0.47 (0.20,1.13)	0.45 (0.18,1.11)
0.45 (0.22,0.94)	2.12 (0.89,5.05)	1.89 (0.58,6.19)	1.28 (0.49,3.32)	1.18 (0.49,2.87)	1.14 (0.38,3.42)	IXE	0.99 (0.39,2.54)	0.69 (0.30,1.60)	0.64 (0.27,1.49)	0.54 (0.24,1.22)	0.52 (0.22,1.20)
0.45 (0.25,0.81)	2.13 (0.95,4.80)	1.90 (0.64,5.68)	1.29 (0.56,2.97)	1.19 (0.56,2.54)	1.15 (0.43,3.11)	1.01 (0.39,2.57)	GOL	0.70 (0.35,1.40)	0.64 (0.31,1.31)	0.54 (0.28,1.06)	0.52 (0.26,1.05)
0.65 (0.44,0.97)	3.06 (1.53,6.12)	2.73 (0.99,7.49)	1.85 (0.90,3.81)	1.71 (0.92,3.20)	1.65 (0.67,4.06)	1.45 (0.62,3.34)	1.44 (0.71,2.90)	ABA	0.92 (0.52,1.64)	0.78 (0.46,1.31)	0.75 (0.43,1.31)
0.71 (0.46,1.08)	3.32 (1.64,6.75)	2.96 (1.07,8.22)	2.00 (0.96,4.20)	1.86 (1.09,3.15)	1.79 (0.72,4.46)	1.57 (0.67,3.68)	1.56 (0.76,3.19)	1.09 (0.61,1.94)	SEC 150 mg	0.85 (0.49,1.46)	0.81 (0.45,1.45)
0.84 (0.59,1.18)	3.93 (2.02,7.62)	3.50 (1.30,9.42)	2.37 (1.18,4.75)	2.20 (1.21,3.97)	2.12 (0.88,5.09)	1.85 (0.82,4.19)	1.84 (0.94,3.61)	1.28 (0.76,2.16)	1.18 (0.69,2.04)	UST	0.96 (0.57,1.62)
0.87 (0.59,1.30)	4.11 (2.05,8.22)	3.66 (1.33,10.06)	2.48 (1.20,5.11)	2.30 (1.23,4.30)	2.22 (0.90,5.45)	1.94 (0.84,4.49)	1.93 (0.95,3.89)	1.34 (0.76,2.35)	1.24 (0.69,2.21)	1.05 (0.62,1.77)	GKM

PBO, placebo; IFN, infliximab; ADA, adalimumab; UST, ustekinumab; GOL, golimumab; ABA, abatacept; SEC, secukinumab; CZP, certolizumab pegol; ETN, etanercept; IXE, ixekizumab; GKM, guselkumab.

Table S3. League table of treatment response: analysis based on change of total radiographic score. The effect sizes are reported as SMD and 95% confidence intervals. Comparisons between treatments should be read from right to left.

Placebo	-0.03 (-0.22,0.16)	-0.16 (-0.33,0.02)	-0.19 (-0.35,-0.03)	-0.23 (-0.44,-0.03)	-0.25 (-0.43,-0.07)	-0.33 (-0.58,-0.09)	-0.33 (-0.50,-0.15)	-0.37 (-0.62,-0.12)	-0.45 (-0.64,-0.26)	-0.51 (-0.78,-0.23)	-0.59 (-0.87,-0.30)
0.03 (-0.16,0.22)	ABA	-0.13 (-0.39,0.13)	-0.16 (-0.41,0.08)	-0.21 (-0.49,0.07)	-0.22 (-0.48,0.04)	-0.31 (-0.62,0.01)	-0.30 (-0.56,-0.04)	-0.34 (-0.65,-0.02)	-0.42 (-0.69,-0.16)	-0.48 (-0.82,-0.14)	-0.56 (-0.90,-0.22)
0.16 (-0.02,0.33)	0.13 (-0.13,0.39)	GKM	-0.03 (-0.27,0.20)	-0.08 (-0.35,0.20)	-0.09 (-0.34,0.16)	-0.17 (-0.48,0.13)	-0.17 (-0.42,0.08)	-0.21 (-0.52,0.10)	-0.29 (-0.55,-0.04)	-0.35 (-0.68,-0.02)	-0.43 (-0.76,-0.10)
0.19 (0.03,0.35)	0.16 (-0.08,0.41)	0.03 (-0.20,0.27)	UST	-0.04 (-0.30,0.22)	-0.06 (-0.30,0.18)	-0.14 (-0.44,0.15)	-0.13 (-0.37,0.10)	-0.18 (-0.47,0.12)	-0.26 (-0.51,-0.02)	-0.31 (-0.63,0.01)	-0.40 (-0.72,-0.07)
0.23 (0.03,0.44)	0.21 (-0.07,0.49)	0.08 (-0.20,0.35)	0.04 (-0.22,0.30)	CZP	-0.02 (-0.29,0.25)	-0.10 (-0.42,0.22)	-0.09 (-0.36,0.18)	-0.13 (-0.46,0.19)	-0.22 (-0.50,0.06)	-0.27 (-0.62,0.07)	-0.35 (-0.70,-0.00)
0.25 (0.07,0.43)	0.22 (-0.04,0.48)	0.09 (-0.16,0.34)	0.06 (-0.18,0.30)	0.02 (-0.25,0.29)	SEC 150 mg	-0.08 (-0.39,0.22)	-0.07 (-0.26,0.11)	-0.12 (-0.42,0.19)	-0.20 (-0.46,0.06)	-0.25 (-0.58,0.07)	-0.34 (-0.67,-0.00)
0.33 (0.09,0.58)	0.31 (-0.01,0.62)	0.17 (-0.13,0.48)	0.14 (-0.15,0.44)	0.10 (-0.22,0.42)	0.08 (-0.22,0.39)	GOL	0.01 (-0.30,0.31)	-0.03 (-0.39,0.32)	-0.12 (-0.43,0.19)	-0.17 (-0.55,0.20)	-0.26 (-0.63,0.12)
0.33 (0.15,0.50)	0.30 (0.04,0.56)	0.17 (-0.08,0.42)	0.13 (-0.10,0.37)	0.09 (-0.18,0.36)	0.07 (-0.11,0.26)	-0.01 (-0.31,0.30)	SEC 300 mg	-0.04 (-0.35,0.27)	-0.13 (-0.38,0.13)	-0.18 (-0.51,0.15)	-0.26 (-0.60,0.07)
0.37 (0.12,0.62)	0.34 (0.02,0.65)	0.21 (-0.10,0.52)	0.18 (-0.12,0.47)	0.13 (-0.19,0.46)	0.12 (-0.19,0.42)	0.03 (-0.32,0.39)	0.04 (-0.27,0.35)	IXE	-0.09 (-0.34,0.17)	-0.14 (-0.51,0.24)	-0.22 (-0.60,0.16)
0.45 (0.26,0.64)	0.42 (0.16,0.69)	0.29 (0.04,0.55)	0.26 (0.02,0.51)	0.22 (-0.06,0.50)	0.20 (-0.06,0.46)	0.12 (-0.19,0.43)	0.13 (-0.13,0.38)	0.09 (-0.17,0.34)	ADA	-0.05 (-0.39,0.28)	-0.14 (-0.48,0.20)
0.51 (0.23,0.78)	0.48 (0.14,0.82)	0.35 (0.02,0.68)	0.31 (-0.01,0.63)	0.27 (-0.07,0.62)	0.25 (-0.07,0.58)	0.17 (-0.20,0.55)	0.18 (-0.15,0.51)	0.14 (-0.24,0.51)	0.05 (-0.28,0.39)	ETN	-0.08 (-0.48,0.31)
0.59 (0.30,0.87)	0.56 (0.22,0.90)	0.43 (0.10,0.76)	0.40 (0.07,0.72)	0.35 (0.00,0.70)	0.34 (0.00,0.67)	0.26 (-0.12,0.63)	0.26 (-0.07,0.60)	0.22 (-0.16,0.60)	0.14 (-0.20,0.48)	0.08 (-0.31,0.48)	IFN

PBO, placebo; IFN, infliximab; ADA, adalimumab; UST, ustekinumab; GOL, golimumab; ABA, abatacept; SEC, secukinumab; CZP, certolizumab pegol; ETN, etanercept; IXE, ixekizumab; GKM, guselkumab.

Table S4. League table of treatment response: analysis based on discontinuation due to adverse events of psoriatic arthritis patient receiving bDMARDs. The effect sizes are reported as odds ratio and 95% confidence intervals. Comparisons between

Placebo	1.71 (0.38,7.62)	1.03 (0.06,16.69)	2.08 (0.45,9.62)	0.49 (0.13,1.84)	4.00 (0.47,33.75)	1.26 (0.20,8.20)	0.45 (0.12,1.70)	0.49 (0.09,2.71)	0.99 (0.20,4.96)	0.33 (0.07,1.54)	0.22 (0.08,0.60)
0.58 (0.13,2.60)	ADA	0.60 (0.03,14.18)	1.22 (0.14,10.32)	0.29 (0.04,2.10)	2.34 (0.17,31.56)	0.74 (0.11,4.79)	0.26 (0.04,1.94)	0.29 (0.03,2.77)	0.58 (0.06,5.20)	0.19 (0.02,1.64)	0.13 (0.02,0.77)
0.97 (0.06,15.73)	1.66 (0.07,39.20)	ETN	2.02 (0.08,48.51)	0.48 (0.02,10.40)	3.88 (0.12,129.65)	1.23 (0.04,35.14)	0.43 (0.02,9.54)	0.48 (0.02,12.52)	0.96 (0.04,24.02)	0.32 (0.01,7.72)	0.21 (0.01,4.06)
0.48 (0.10,2.22)	0.82 (0.10,6.99)	0.50 (0.02,11.89)	CZP	0.24 (0.03,1.78)	1.92 (0.14,26.55)	0.61 (0.05,6.81)	0.22 (0.03,1.64)	0.24 (0.02,2.34)	0.48 (0.05,4.40)	0.16 (0.02,1.39)	0.10 (0.02,0.65)
2.03 (0.54,7.60)	3.48 (0.48,25.51)	2.10 (0.10,45.65)	4.23 (0.56,31.92)	SEC 300 mg	8.14 (0.66,99.82)	2.57 (0.26,25.32)	0.91 (0.14,5.95)	1.00 (0.12,8.64)	2.01 (0.25,16.16)	0.67 (0.11,4.05)	0.44 (0.08,2.32)
0.25 (0.03,2.11)	0.43 (0.03,5.78)	0.26 (0.01,8.60)	0.52 (0.04,7.19)	0.12 (0.01,1.51)	IFN	0.32 (0.02,5.39)	0.11 (0.01,1.39)	0.12 (0.01,1.89)	0.25 (0.02,3.59)	0.08 (0.01,1.14)	0.05 (0.01,0.57)
0.79 (0.12,5.13)	1.35 (0.21,8.78)	0.81 (0.03,23.32)	1.65 (0.15,18.44)	0.39 (0.04,3.83)	3.16 (0.19,53.92)	IXE	0.35 (0.04,3.52)	0.39 (0.03,4.89)	0.78 (0.07,9.24)	0.26 (0.02,2.94)	0.17 (0.02,1.43)
2.23 (0.59,8.49)	3.82 (0.52,28.35)	2.30 (0.10,50.52)	4.65 (0.61,35.47)	1.10 (0.17,7.17)	8.94 (0.72,110.66)	2.82 (0.28,28.09)	GOL	1.10 (0.13,9.60)	2.21 (0.27,17.95)	0.74 (0.10,5.66)	0.48 (0.09,2.58)
2.03 (0.37,11.20)	3.48 (0.36,33.61)	2.09 (0.08,54.91)	4.23 (0.43,41.90)	1.00 (0.12,8.63)	8.13 (0.53,124.88)	2.57 (0.20,32.30)	0.91 (0.10,7.95)	GKM	2.01 (0.19,21.06)	0.67 (0.07,6.68)	0.44 (0.06,3.20)
1.01 (0.20,5.06)	1.73 (0.19,15.55)	1.04 (0.04,25.98)	2.10 (0.23,19.41)	0.50 (0.06,3.98)	4.04 (0.28,58.50)	1.28 (0.11,15.06)	0.45 (0.06,3.67)	0.50 (0.05,5.19)	ABA	0.33 (0.04,3.09)	0.22 (0.03,1.47)
3.04 (0.65,14.19)	5.20 (0.61,44.47)	3.13 (0.13,75.50)	6.32 (0.72,55.52)	1.49 (0.25,9.02)	12.15 (0.87,168.82)	3.84 (0.34,43.31)	1.36 (0.18,10.46)	1.49 (0.15,14.90)	3.01 (0.32,27.99)	SEC 150 mg	0.65 (0.10,4.16)
4.64 (1.67,12.84)	7.94 (1.30,48.40)	4.78 (0.25,92.73)	9.65 (1.53,60.72)	2.28 (0.43,12.06)	18.55 (1.75,197.16)	5.86 (0.70,49.27)	2.08 (0.39,11.14)	2.28 (0.31,16.65)	4.59 (0.68,30.92)	1.53 (0.24,9.69)	UST

treatments should be read from right to left.

PBO, placebo; IFN, infliximab; ADA, adalimumab; UST, ustekinumab; GOL, golimumab; ABA, abatacept; SEC, secukinumab; CZP, certolizumab pegol; ETN, etanercept; IXE, ixekizumab; GKM, guselkumab.

Figure S1. Risk of bias, presented with the Cochrane Collaboration's Assessment Tool.

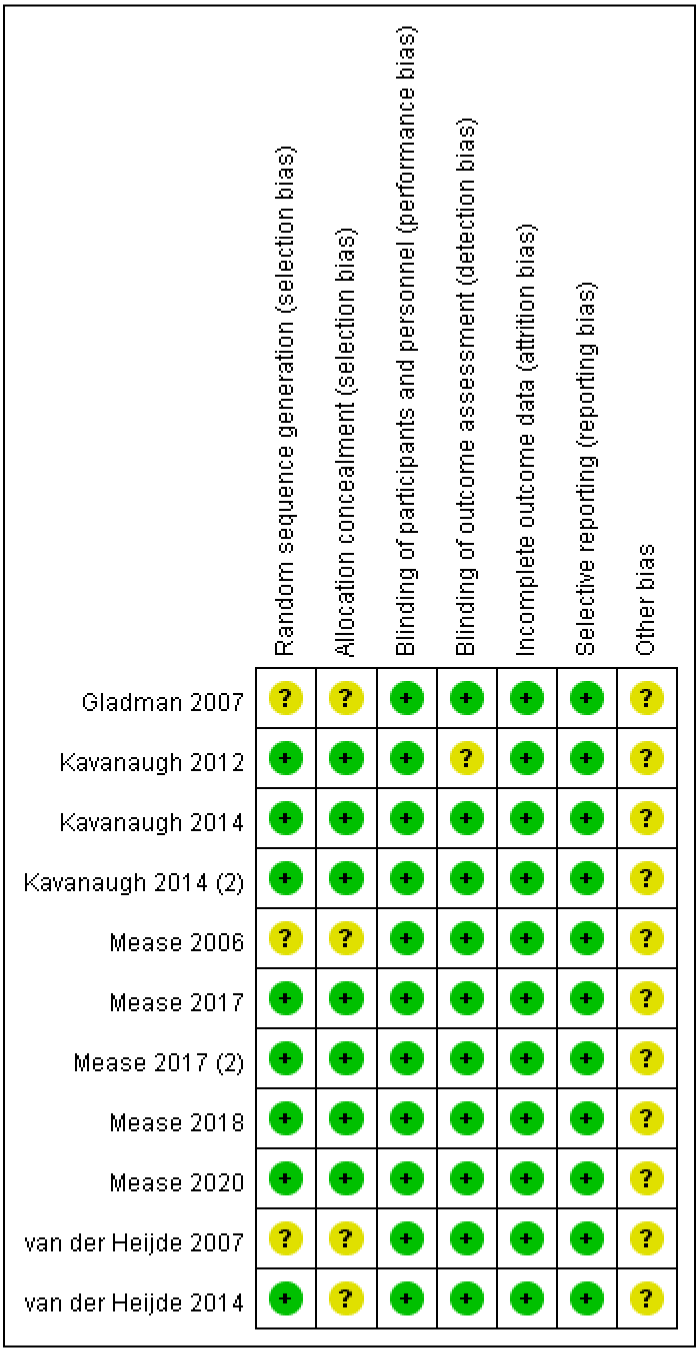

Figure S2. Funnel plot for radiographic non-progression of psoriatic arthritis patient receiving bDMARDs

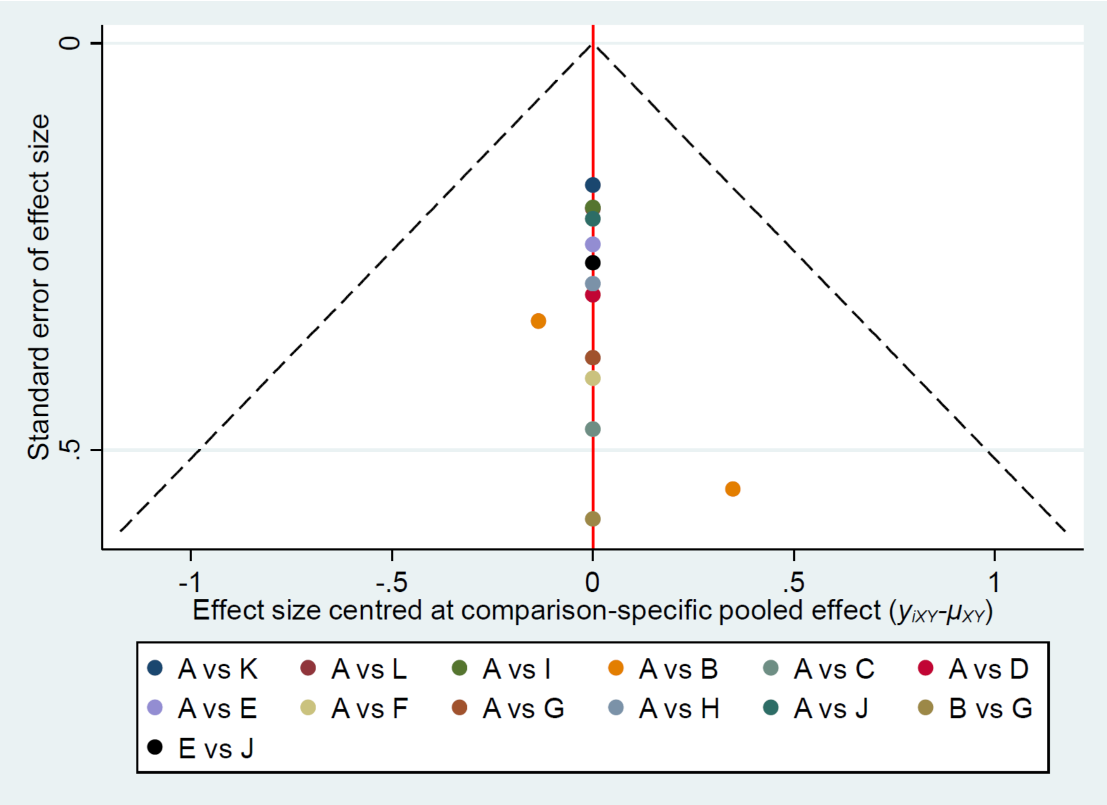

A, Placebo; B, Adalimumab; C, Etanercept; D, Certolizumab pegol; E, Secukinumab 300mg; F, Infliximab; G, Ixekizumab; H, Golimumab; I, Abatacept, J, Secukinumab 150mg; K, Ustekinumab; L, Guselkumab

Figure S3. Funnel plot for change of total radiographic score of psoriatic arthritis patient receiving bDMARDs

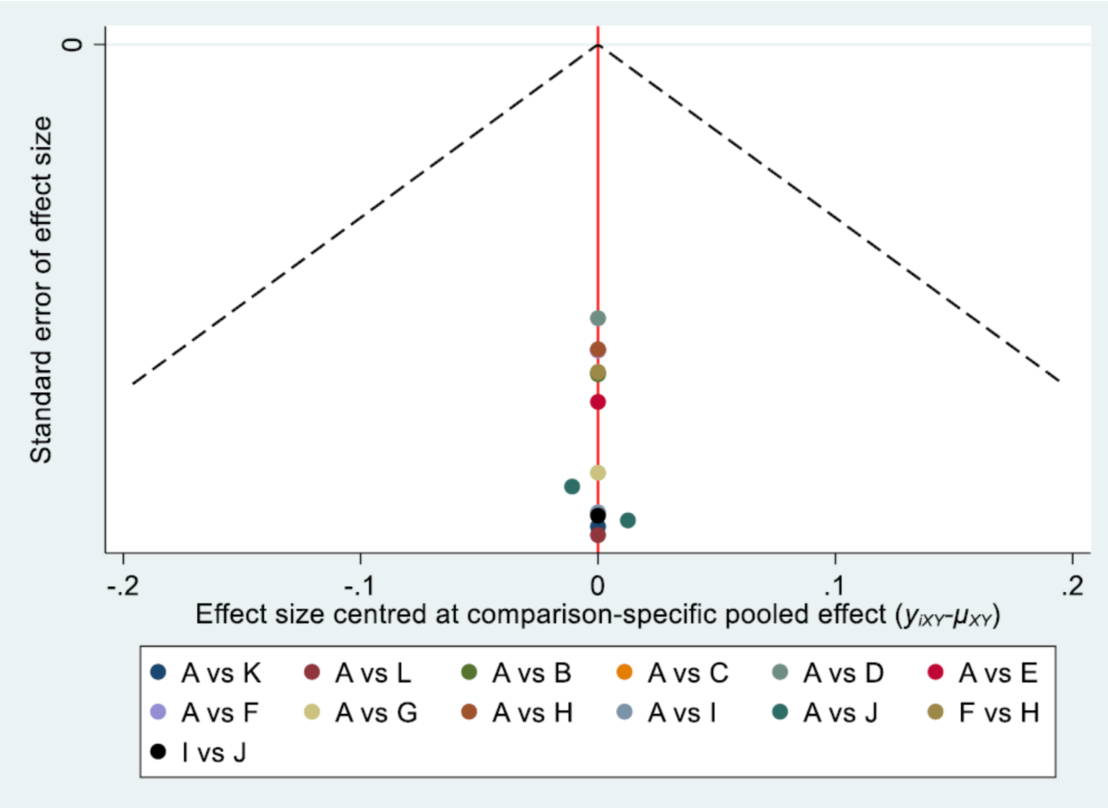

A, Placebo; B, Abatacept; C, Guselkumab; D, Ustekinumab; E, Certolizumab pegol; F, Secukinumab 150mg; G, Golimumab; H, Secukinumab 300mg; I, Ixekizumab; J, Adalimumab; K, Etanercept; L, Infliximab

Figure S4. Funnel plot for discontinuation due to adverse events of psoriatic arthritis patient receiving bDMARDs

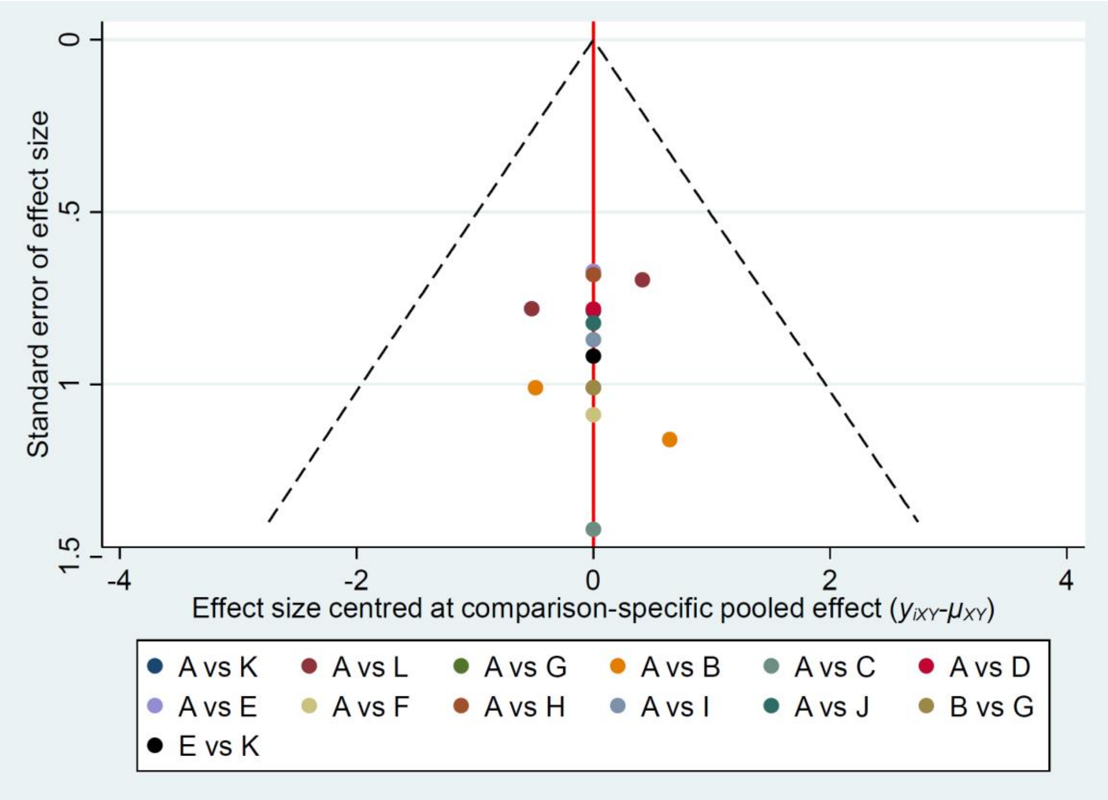

A, Placebo; B, Abatacept; C, Etanercept; D, Certolizumab pegol; E, Secukinumab 300mg; F, Infliximab; G, Ixekizumab; H, Golimumab; I, Guselkumab; J, Abatacept; K, Secukinumab 150mg; L, Ustekinumab

Figure S5. Egger's test for radiographic non-progression

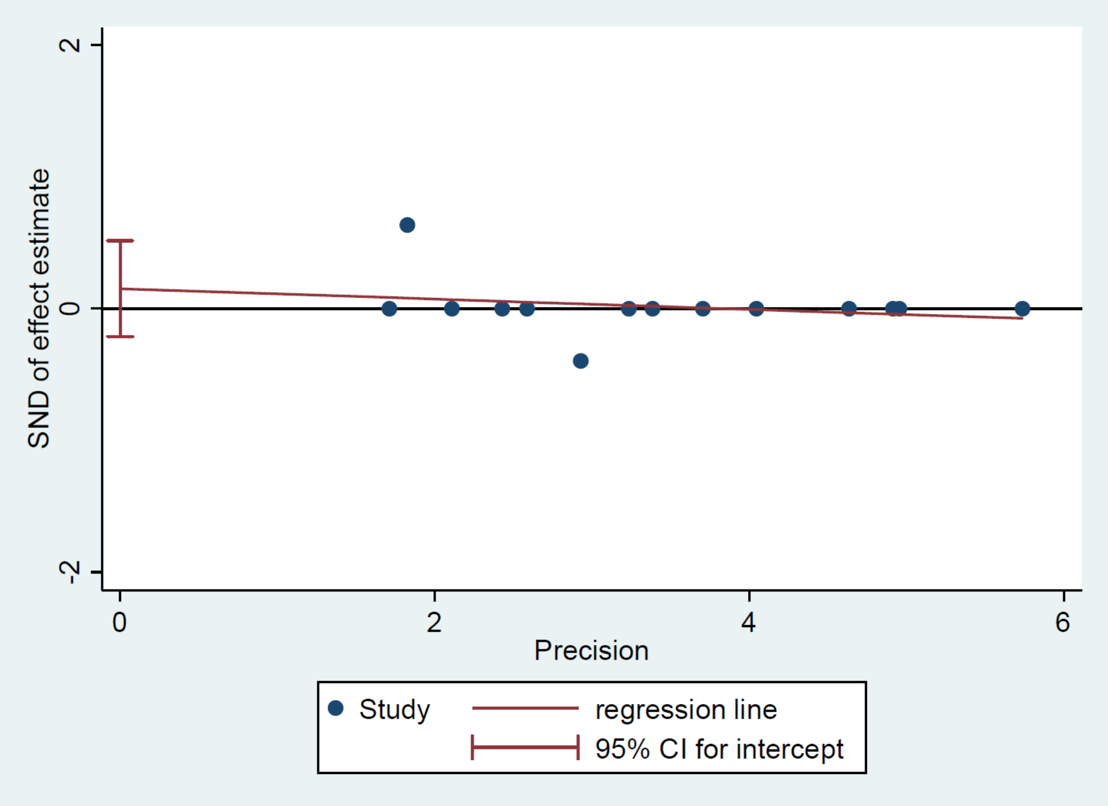

Figure S6. Egger's test for change of total radiographic score

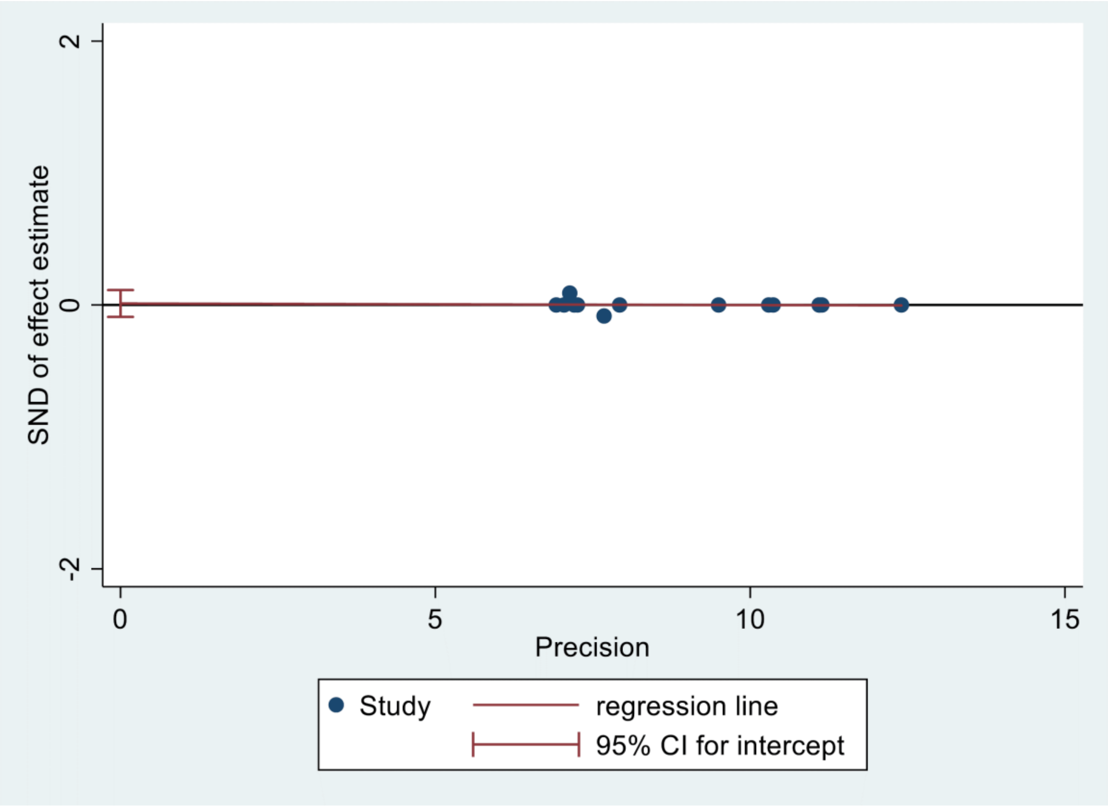

Figure S7. Egger's test for discontinuation due to adverse events.

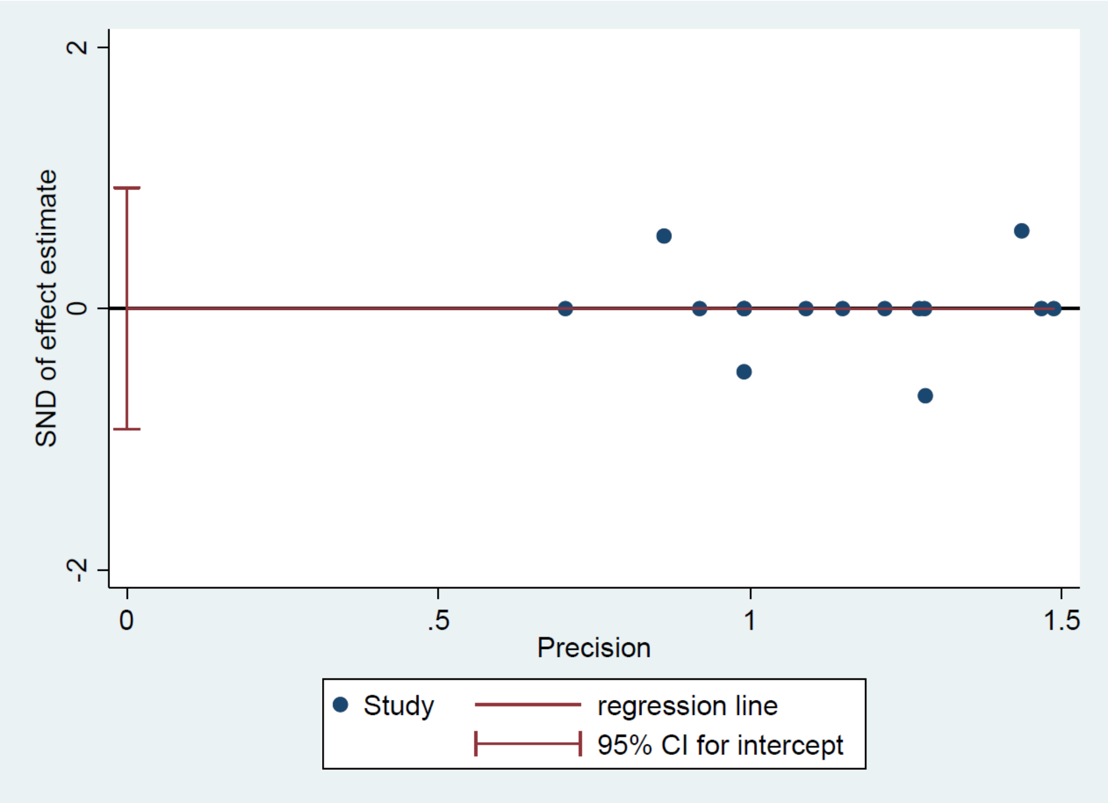

Supplement: Supplementary file 1 [file pharmaceutics-14-02140-s001.zip › pharmaceutics-1915622-supplementary.pdf]
